# *Plasmodium* Strain Determines Dendritic Cell Function Essential for Survival from Malaria

**DOI:** 10.1371/journal.ppat.0030096

**Published:** 2007-07-06

**Authors:** Michelle N Wykes, Xue Q Liu, Lynette Beattie, Danielle I Stanisic, Katryn J Stacey, Mark J Smyth, Ranjeny Thomas, Michael F Good

**Affiliations:** 1 The Queensland Institute of Medical Research, Brisbane, Queensland, Australia; 2 Immunology and Infection Unit, University of York, York, United Kingdom; 3 Infection and Immunity Division, The Walter and Eliza Hall Institute of Medical Research, Parkville, Victoria, Australia; 4 Institute for Molecular Bioscience, CRC for Chronic Inflammatory Diseases, University of Queensland, Brisbane, Queensland, Australia; 5 Cancer Immunology Program, Peter MacCallum Cancer Centre, East Melbourne, Victoria, Australia; 6 Centre for Immunology and Cancer Research, University of Queensland, Woolloongabba, Queensland, Australia; Institut Pasteur, France

## Abstract

The severity of malaria can range from asymptomatic to lethal infections involving severe anaemia and cerebral disease. However, the molecular and cellular factors responsible for these differences in disease severity are poorly understood. Identifying the factors that mediate virulence will contribute to developing antiparasitic immune responses. Since immunity is initiated by dendritic cells (DCs), we compared their phenotype and function following infection with either a nonlethal or lethal strain of the rodent parasite, *Plasmodium yoelii,* to identify their contribution to disease severity. DCs from nonlethal infections were fully functional and capable of secreting cytokines and stimulating T cells. In contrast, DCs from lethal infections were not functional. We then transferred DCs from mice with nonlethal infections to mice given lethal infections and showed that these DCs mediated control of parasitemia and survival. IL-12 was necessary for survival. To our knowledge, our studies have shown for the first time that during a malaria infection, DC function is essential for survival. More importantly, the functions of these DCs are determined by the strain of parasite. Our studies may explain, in part, why natural malaria infections may have different outcomes.

## Author Summary

Malaria is a complex disease and there is little data on why *Plasmodium* infections have different outcomes that can range from asymptomatic to lethal infections. Since immunity is initiated by dendritic cells (DCs), several studies have investigated DC function during malaria. Current data on the effects of infection on DC functions are inconclusive, with one school of thought being that DC function is normal and the other that DC function is compromised. However, these studies have used different species and strains of *Plasmodium*. We have compared DC function during a lethal and nonlethal infection and found significant differences. Our study shows that the strain of parasite determines if DCs remain functional following infection. Moreover, by transferring DCs between mice, we show that DC function is essential for survival from a lethal infection. This study offers some insight into the current controversy and offers a plausible explanation for differences in the severity of disease.

## Introduction

The factors that determine the virulence of *Plasmodium* are poorly understood. While adhesion of parasitized red blood cells (pRBCs) to the vascular endothelium in the brain [1–4], placenta [5–7], and other tissues is believed to be critical, inflammatory cytokines have been implicated in both human and rodent diseases [8–18]. Inappropriate cellular responses [19–23] or loss of immune cells during malaria [24–29] are also implicated in poor outcome. In particular, a recent study found that CD8^+^ dendritic cells (DCs) (only found in mice) die by apoptosis during rodent malaria [30].

DCs initiate immune responses and were first implicated in the pathogenesis of human malaria when in vitro studies found that Plasmodium falciparum–infected red cells could block lipopolysaccharide (LPS)-induced maturation of DCs in culture [31]. This was mediated by binding of the pRBCs to CD36 on DCs [32]. However, murine studies show that DCs can phagocytose P. chabaudi blood-stage pRBCs [33] and present pRBC-derived antigens to CD4^+^ T cells to initiate the development of protective Th1-dependent immune responses [33,34]. These responses are generated by CD8^−^ DCs during the acute phase of infection [30]. However, DCs from infected mice are unable to prime CD8^+^ T cells to proliferate and secrete cytokines [34,35], which impairs cross-presentation of viral antigens [36]. It has been proposed that hemozoin, rather than infected erythrocyte membranes, impairs murine DC function [37], but another study suggests that hemozoin activates innate immune responses in mice by a toll-like receptor (TLR9)-mediated, MyD88-dependent, but chloroquine-sensitive mechanism [38]. In contrast, other studies have found that DCs from mice infected with P. chabaudi or P. yoelii were fully functional [39–41].

Although DCs have been shown to be important for initiating immunity to malaria [30,34–36,42,43], studies describing the function of DCs following infection have not explored the direct role of DCs in affecting disease outcome (i.e., survival, anaemia, or parasitemia). Furthermore, there is little data on why *Plasmodium* infections have different outcomes that can range from asymptomatic to lethal infections. On the basis of previous studies, we hypothesized that lethal parasites may compromise DC function [34–36], while DCs from nonlethal infections were fully functional and able to mediate immune responses and survival from malaria [39–41]. As such, we compared DC phenotype and function in response to lethal and nonlethal strains of the rodent parasite P. yoelii to correlate DC function with disease outcome. Splenic DCs were investigated, as the spleen has been shown to be a major site of parasite killing and regulation of parasite-specific immune responses [29,44,45]. Our studies show that DCs from mice with nonlethal infections are fully functional, while DC function is perturbed during lethal infections. The functional DCs from nonlethal infections can protect mice from lethal infections, demonstrating that DCs contribute substantially to the control of parasitemia and mediate survival from malaria.

## Results

### Comparison of Parasitemias during Lethal and Nonlethal Infections

To establish the course of lethal and nonlethal *Plasmodium* infections, cohorts of C57Bl/6 mice were infected with either nonlethal *P. yoelii 17XNL* or lethal *P. yoelii YM,* and parasitemia was monitored over 32 d. Since preliminary studies (unpublished data) established that the two parasite strains had different growth rates, mice were given either 10^5^ nonlethal or 10^4^ lethal pRBCs so that all mice would develop patent (detectable) parasitemia at approximately the same time (Figure 1, day 4). We then studied DC function after the first week of infection, when adaptive immune responses are initiated and changes to the spleen are consistent. Since the parasites had very different growth curves, we could not achieve similar parasitemias at the time of testing.

**Figure 1:**
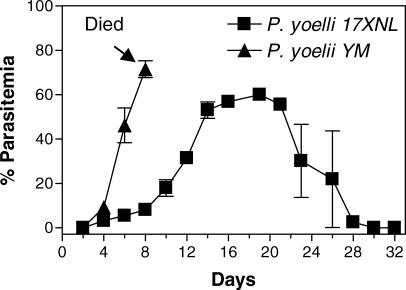
Comparison of Parasitemias Groups of three mice were infected with nonlethal *P. yoelii 17XNL* or lethal P *yoelii YM*. Blood smears were made every 2–3 d and stained with Giemsa. The numbers of infected red cells were counted in at least 20 fields of approximately 150 red cells to establish the absence of patent parasitemia. Parasitemias were monitored in every experiment, and this figure is an example of the typical course of infection. Error bars represent mean parasitemia ± standard error of the mean. The parasitemia curves represent one of multiple experiments.

### Comparison of DC Surface Phenotype during Lethal and Nonlethal Infections

Previous studies of mice infected with nonlethal P. yoelii found that DCs had an immature phenotype with lower levels of MHC class II, CD80, and CD86 expression [35]. However, studies have not determined if DC phenotype or maturation differ between lethal and nonlethal P. yoelii infections. As such, mice were infected with either nonlethal *P. yoelii 17XNL* or lethal *P. yoelii YM,* and the maturation of CD11c^+^ DCs was analysed in infected and naïve mice after 6.5 d. No significant differences in MHC class II, CD80, or CD86 expression by CD11c^+^ cells were detected between the two strains (Figure 2A and 2B), although the DCs from infected mice were relatively immature compared to DCs from naïve mice.

**Figure 2:**
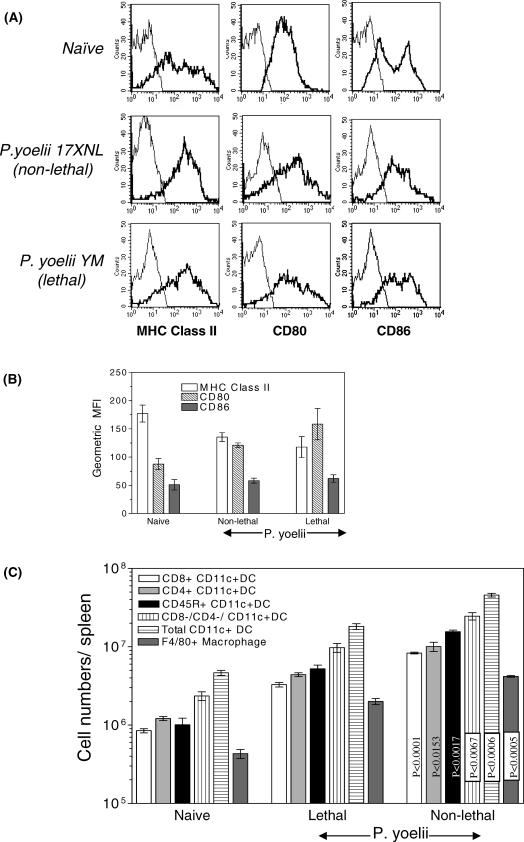
Maturation Profiles and Phenotype of DCs following Infection with Lethal and Nonlethal P. yoelii Groups of three mice were infected with either nonlethal *P. yoelii 17XNL* or lethal *P. yoelii YM*. DCs were isolated from individual spleens and labelled to detect MHC class II, CD80, and CD86 by flow cytometry 7 d after infection. (A) The flow cytometry profiles are examples of DCs from naïve mice or infected mice expressing the relevant cell surface molecules. The profiles were similar between three experiments. (B) The bar charts represent the average mean fluorescence intensity (MFI) of DCs expressing the relevant cell surface molecules from at least three mice per group. The error bars represent the MFI ± standard error of the mean. The bar charts represent one of three experiments that gave similar profiles. (C) Bar chart shows the absolute numbers of CD11c^+^CD8^+^ DCs, CD11c^+^CD4^+^ DCs, CD11c^+^CD4^−^CD8^−^ DCs, CD11c^+^CD45R(B220)^+^ DCs, or F480^hi^ macrophages per spleen in infected and naïve mice for groups of mice ± standard error of the mean. The absolute number of cells for each subpopulation was calculated by multiplying the percentage of cells by the total number of cells isolated from the spleen. Labelling by the control antibody was used to set the gates. The *p*-values in the bars for a nonlethal infection represent the statistical difference compared to a lethal infection.

We then enumerated the number of CD11c^+^ DCs per spleen following both infections and found that *P. yoelii YM* and *P. yoelii 17XNL* infections induced 3.5- and 10-fold increases, respectively, in the total numbers of CD11c^+^ DCs in the spleen compared to naïve mice (Figure 2C). We next examined DC subpopulations in infected and naïve mice and observed that the following subsets were more prevalent in mice following infection with the nonlethal strain compared to the lethal strains: CD45R(B220)^+^ DCs (3-fold); CD8^+^ DCs (2.8-fold); CD4^+^ DCs (2.3-fold); and CD4^−^/CD8^−^ DCs (2.5-fold). F4/80^hi^ macrophages were also more prevalent in mice infected with the nonlethal strain (4.7-fold). These differences were all statistically significant, and the *p*-values are noted in the figure. These studies highlighted that nonlethal infections stimulated a greater influx of DCs into the spleen, which correlated with survival compared to lethal infections where the DC influx was less pronounced.

### Comparison of Inflammatory Cytokines during Lethal and Nonlethal Infections

Interleukin-12 (IL-12) is produced by CD8^+^ and B220^+^ DCs to mediate adaptive immune responses, and tumour necrosis factor (TNF)-α produced by several sources mediates DC maturation. To investigate differences in these cytokines between lethal and nonlethal infections, mice were bled at days 0, 4, and 7 after infection, and the levels of inflammatory cytokines TNF-α and IL-12 were measured in the serum (Figure 3A). TNF-α levels were increased following both infections, and the differences in levels between lethal and nonlethal P. yoelii infections, 4 and 7 d after infection were not significant (*p* > 0.5). In contrast, mice infected with a nonlethal *P. yoelii 17XNL* infection had significantly 10- to 57-fold more pg/ml IL-12 in their serum compared to naïve mice or mice given a lethal infection (Figure 3A).

**Figure 3:**
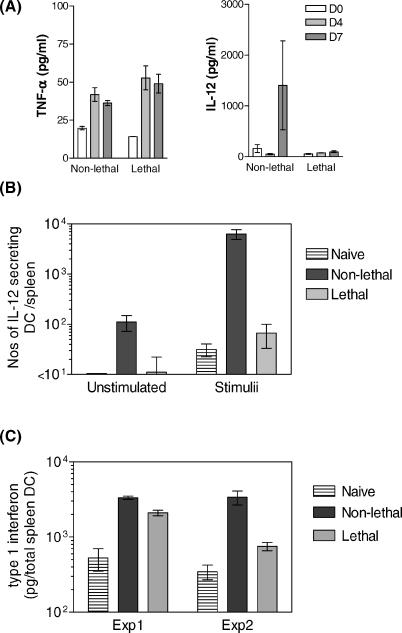
Comparison of Cytokine Profiles from Groups of Mice following Nonlethal and Lethal P. yoelii Infections and Cytokine Secretion by DCs (A) Groups of three mice were infected with nonlethal *P. yoelii 17XNL* or lethal *P. yoelii YM*. After 4 and 7 d, the levels of IL-12 and TNF-α were assessed in the serum using the Becton Dickinson bead array assay. Error bars represent the pg of cytokine per ml ± standard error of the mean. The data represent one of duplicate experiments. (B) Groups of three or five mice were infected with nonlethal or lethal *P. yoelii.* After 7 d, total CD11c^+^ DCs were isolated from individual, naïve, or infected mice, cultured with or without CpG oligonucleotide plus LPS overnight, and then tested for IL-12 secretion by a p70 IL-12–specific ELISPOT assay. Error bars represent the mean number of DCs secreting IL-12 per spleen ± standard error of the mean. The data represent one of multiple experiments. (C) Total CD11c^+^ DCs were isolated from groups of three individual, naïve, or infected mice and cultured with CpG oligonucleotide and poly-I:C for 36 h. The supernatants were tested for secretion of α-interferon by ELISA. Error bars represent the mean pg secretion of α-interferon per spleen ± standard error of the mean. The data represent one of triplicate experiments for the nonlethal parasite and duplicate for the lethal parasite.

IL-12 has been implicated as an important cytokine in resistance to malaria [9,13,14,46–48]. To assess whether DCs secrete biologically active IL-12 following infection, total CD11c^+^ DC populations were isolated from infected and naïve mice and p70 IL-12 production was assessed using an ELISPOT assay (Figure 3B). The numbers of DCs secreting IL-12 without in vitro stimulation were negligible in all mice except those infected with *P. yoelii 17XNL.* Following in vitro stimulation with CpG and LPS (chosen to maximally stimulate IL-12 production by all DC subpopulations via TLR9 and TLR4 respectively) [49–52], the number of IL-12-producing DCs from mice infected with nonlethal *P. yoelii 17XNL* was more than 200-fold higher than DCs from naïve mice (*p* < 0.0004) and 94-fold higher than DCs from mice infected with lethal *P. yoelii YM* (*p* < 0.0004).

Finally, we measured the secretion of α-(type1)-interferon by DCs since (a) B220^+^ DCs secrete this cytokine, (b) mice with nonlethal infections had 3-fold more B220^+^ DCs than mice with lethal infections, and (c) since it is suggested to improve priming of myeloid DCs [53–55]. DCs from lethal and nonlethal infections were cultured with CpG DNA and poly IC [49,56], and the secretion of α-(type 1)-interferon was assessed by ELISA (Figure 3C). We found that 5- to 10-fold more α-(type 1)-interferon was produced per spleen by DCs from nonlethal infected mice compared to naïve mice (*p* < 0.0015), consistent with the abundance of B220^+^ DCs. Mice with nonlethal infections only produced 1.5- to 4-fold more α-(type 1)-interferon than DCs from lethal infections (*p* < 0.0135). The DCs from lethal infections produced, however, significantly more α-(type 1)-interferon per spleen than naïve mice (*p* < 0.0012), indicating that B220^+^ DCs could secrete this cytokine during both infections.

### Comparison of DC Functions following Infection

DCs were then compared functionally from mice infected with lethal and nonlethal P. yoelii. CD11c^+^ DCs were isolated by immuno-magnetic positive selection from groups of infected (day 6.5) and naïve C57BL/6 mice, and varying numbers were cultured with a fixed number of allogeneic T cells (BALB/c), or syngeneic T cells from ovalbumin-specific T cell receptor transgenic (OTII) mice in the presence of ovalbumin (Figure 4). CD11c^+^ DCs from mice infected with nonlethal *P. yoelii 17XNL* were better able to stimulate proliferation of allogeneic T cells or present ovalbumin to OTII T cells compared to DCs from mice infected with lethal *P. yoelii YM* (Figure 4). DCs from naïve mice were more efficient antigen-presenting cells than DCs from mice infected with either parasite.

**Figure 4:**
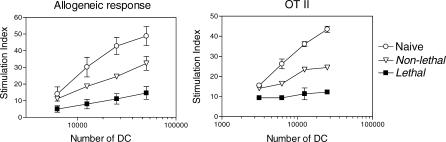
Comparison of the Antigen Presentation Function of CD11c^+^ DCs Isolated from Groups of Mice following Nonlethal and Lethal P. yoelii Infections Groups of three mice were infected with nonlethal or lethal P. yoelii. After 7 d, CD11c^+^ DCs were isolated from individual mice, serially diluted, and incubated with a fixed number of allogeneic T cells (BALB/c) or syngeneic T cells from OTII mice with ovalbumin. After 3 d, the cultures were pulsed with ^3^H-thymidine for 18 h and the uptake of radiolabel measured. The error bars represent mean count ± standard error of the mean. These assays were undertaken three times.

### Comparison of DCs in Mediating Survival from Lethal Infections

Since we observed significant differences in DC function between lethal and nonlethal infections, we adoptively transferred DCs from infected mice to naïve mice to determine whether DCs could modulate the outcome of infection in recipient mice. CD11c^+^ DCs were isolated from naïve mice or mice infected with either lethal *P. yoelii YM* or nonlethal *P. yoelii 17XNL.* The infected mice were drug cured before the isolation of DCs to prevent the transfer of parasites from donor mice. Approximately 1.5 × 10^7^ DCs from each group were then transfused into naïve mice, which were then infected with lethal *P. yoelii YM,* 24 h later. Mice given DCs from naïve mice or from mice infected with *P. yoelii YM* died by the eighth day (Figure 5A). In contrast, approximately 80% of mice given DCs isolated from mice infected with nonlethal *P. yoelii 17XNL* survived for at least 18 d, and 20% cleared the infection. In repeat experiments, with similar survival results, we also observed that the hemoglobin levels dropped after 8–10 d, and parasitemia levels increased but peaked at approximately 40%–50% if mice received DCs from mice previously infected with *P. yoelii 17XNL* (Figure 5A). The difference in survival between mice given DCs from nonlethal infections compared to DCs from naïve mice (*p* < 0.0082) or DCs from lethal infections (*p* < 0.0031) was statistically significant. These experiments comparing DCs from *P. yoelii 17XNL*–infected and naïve mice were repeated twice with 1.5 × 10^7^ DCs and once with 8 × 10^6^ DCs, with similar results

**Figure 5:**
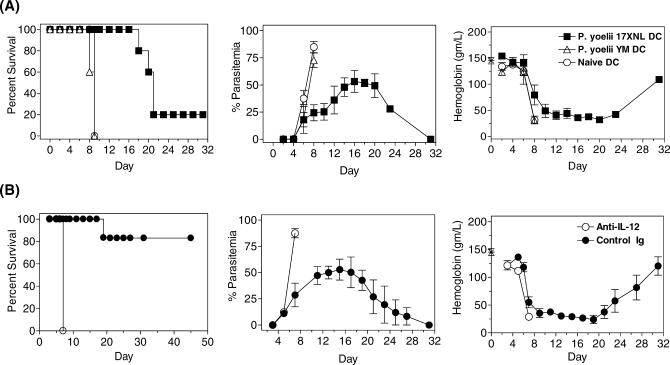
Protection Is Mediated by DCs and by IL-12 (A) To measure the protection offered by CD11c^+^ DCs, large groups of mice were infected with nonlethal *P. yoelii 17XNL* or lethal *P. yoelii YM*. DCs were isolated from naïve or infected (drug-cured) mice, and 1.5 × 10^7^ DCs were transferred to each naïve mouse in groups of four or five. After 24 h, each mouse was infected with a lethal dose of *P. yoelii YM,* and survival, parasitemia, and hemoglobin levels monitored every 1–3 d. This experiment was repeated twice with mice given 1.5 × 10^7^ DCs. (B) To determine the role of IL-12 in DC-mediated protection, groups of mice were infected with nonlethal *P. yoelii 17XNL,* total CD11c^+^ DCs were isolated from these infected mice after drug curing, and 1.5 × 10^7^ DCs were transferred to naïve mice in groups of four or five. The cohorts of mice were given control Ig or anti-IL-12 15 h after the transfer of DCs, and after 24 h, each mouse was infected with a lethal dose of *P. yoelii YM*. Additional doses of control immunoglobulin or anti-IL-12 were given after 3 and 6 d. This experiment was repeated twice. In all experiments, mice were monitored daily following infection and culled if required, but the hemoglobin and parasitemia levels were measured every 2–3 d. Error bars represent ± standard error of the mean. The mean hemoglobin level in 20 naïve mice is represented by a cross-symbol on the *y*-axis.

We next investigated whether DCs mediated survival from lethal infections via IL-12 (Figure 5B). DCs were isolated from mice infected with *P. yoelii 17XNL* and administered to naïve mice (1.5 × 10^7^ DCs per mouse) as described above. After 15 h, these mice were given anti-IL-12 or control-rat immunoglobulin (see Materials and Methods) and infected with *P. yoelii YM* 6–9 h later. Mice were given additional doses of anti-IL-12 or control immunoglobulin 3 and 6 d after infection. All mice given anti-IL-12 died within 8 d. In contrast, 100% of mice given the control immunoglobulin survived for 18 d, and 80% of mice cleared infection. The difference in survival between these groups was statistically significant (*p* < 0.0009).

We finally confirmed that survival from lethal infections was mediated by IL-12 produced by the donor DCs, and not by the recipient mice, by transferring DCs from IL-12 knockout (KO) mice. As such, DCs were isolated from wild-type C57Bl/6 or IL-12KO mice (on a C57Bl/6 background) infected with *P. yoelii 17XNL* and administered to naïve mice (1.5 × 10^7^ DCs per mouse) as described above. The next day, these mice were infected with *P. yoelii YM* and monitored daily. A total of two-thirds of mice given DCs from IL-12KO mice died within 8 d, and the remaining mice died by the 15th day (Figure 6). In contrast, more than 83% of mice given DCs from wild-type mice survived for 22 d, and 50% of these mice cleared infection. The difference in survival between these groups was statistically significant (*p* < 0.0023).

**Figure 6:**
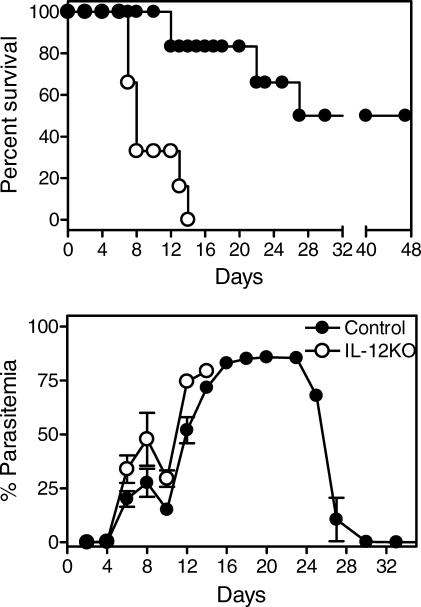
Protection Is Mediated by IL-12 Secreted by Donor DCs To confirm that IL-12 secreted by the donor DC-mediated protection, groups of six C57BL/6 and IL-12KO mice were infected with nonlethal *P. yoelii 17XNL,* total CD11c^+^ DCs were isolated from these infected mice after drug curing, and 1.5 × 10^7^ DCs were transferred to groups naïve mice. After 24 h each mouse was infected with a lethal dose of *P. yoelii YM*. In all experiments, mice were monitored daily following infection and culled if required, but parasitemia levels were measured every 2–3 d. Error bars represent ± standard error of the mean. This experiment was repeated twice.

These studies showed that DCs from lethal infections, previously shown to be unable to produce IL-12 (Figure 3) or present antigen to T cells (Figure 4), were unable to mediate survival from lethal disease. However, the DCs from nonlethal infections, previously shown to be functional (Figures 3 and 4), were able to mediate survival from a lethal disease via secretion of IL-12.

## Discussion

DCs are essential for initiating adaptive and innate immune responses in malaria. Recent studies have shown that CD8^−^ DCs isolated at the acute phase of a nonlethal P. chabaudi infection can stimulate Ag-specific T cell responses [30]. In contrast, DCs from lethal P. berghei–infected mice are unable to prime CD8 T cells [34,35] or cross-present viral antigens during malaria [36]. Numerous studies have shown that IL-12 is an important cytokine in resistance to malaria [9,13,14,46–48], but the role of DCs in IL-12 production is not clear. Most studies to date have focussed on the direct effects of the infection on immunity to malaria (e.g., loss of CD8^+^ T cell priming) or have identified cellular responses that mediate protection (e.g., T cells are required to clear the parasite). However, to our knowledge, no study to date has explained why different infections have different outcomes or directly implicated DCs in mediating survival from malaria. To address this issue, we compared two strains of P. yoelii with different outcomes (lethal and nonlethal) and found that the strain of parasite determines the phenotype and function of DCs that mediate survival from a lethal infection using IL-12.

Previous studies have shown that IL-12 production can be initiated within 2 d of an infection [47], and Th1 responses that predominate initially during infection can be protective [13,14,46–48,57–59]. We now show that DCs are a significant source of IL-12 needed to clear infection, since mice infected with the nonlethal *P. yoelii 17XNL* had nearly 100-fold more IL-12-producing DCs than mice given the lethal *P. yoelii YM*. Furthermore, when DCs from nonlethal infections were transferred to naïve mice that were then infected with a lethal strain, the mice survived significantly longer. Protection was shown to be mediated by IL-12 since depleting IL-12 blocked survival. The donor DCs were a major source of IL-12 as DCs from naïve mice or mice with lethal infections did not secrete IL-12 in vitro (Figure 2), and transfer of DCs from IL-12KO mice confirmed that donor DCs were the source of IL-12. Together the data suggest that nonlethal parasites can induce the development of IL-12-secreting DCs, which mediates protection and survival. In contrast, the failure of lethal parasites to induce significant IL-12 production by DCs is associated with a poor outcome for the host.

Our studies also found that the level of T cell proliferation induced by DCs from mice infected with *P. yoelii 17XNL* nonlethal infections was significant but was not as high as that induced by DCs from naïve mice. This observation possibly reflects the high numbers of CD45R^+^ DCs found during nonlethal infections, which are generally not efficient at inducing proliferation of T cells [49,60]. The substantially diminished ability of DCs from mice infected with *P. yoelii YM* to induce T cell responses suggests that the cell-mediated immune response of mice following *P. yoelii YM* infection could be diminished in vivo and may contribute to the poor outcome.

In conclusion, our studies have shown that following a *Plasmodium* infection the strain of parasite affects the nature and function of the DC response and that this response is a key determinant of survival. Our data suggest that while nonlethal parasites can induce the accumulation of functional DCs in the spleen that can mediate survival from infection, the lethal parasite induces a block in DC function. We thus speculate that lethal malaria results from a failure of DC function as well as a block in the up-regulation of Th1 immune responses. Thus this study may explain why infections can have different outcomes.

## Materials and Methods

### Animals.

Specific pathogen-free, 6–8 wk old, female C57BL/6J mice were obtained from the Animal Resources Centre (http://www.sabc.murdoch.edu.au/), and OTII mice were kindly provided by William Heath (The Walter and Eliza Hall Institute of Medical Research, http://www.wehi.edu.au/). These studies have been reviewed and approved by the Queensland Institute of Medical Research Animal Ethics Committee.

### Infection of mice for flowcytometry.

Cohorts of three to six mice were infected intravenously with 10^5^ *P. yoelii 17XNL* or 10^4^ *P. yoelii YM* pRBCs, and spleens taken after 6.5 d for flow cytometry analysis as preliminary studies found that changes to the DCs were not clear before this time with both strains of parasite.

### Isolation of splenic DCs.

Spleens from naïve or infected C57BL/6J mice were digested as previously described [61,62] and DCs isolated using either anti-CD11c MACS beads according to the manufacturer's instructions (Miltenyi Biotec, http://www.miltenyibiotec.com/). The labelled cell preparations were passed through three to five columns until the purity was >95%. The isolated DCs were always labelled with anti-CD11c-PE for FACS analysis to confirm purity. Viability was assessed by trypan blue or labelling with 7-actinomycin D.

### Flowcytometry of DCs.

To accurately quantify cell numbers shown in Figure 3, the spleens of mice were digested to release DCs and labelled directly. For these studies, approximately 2 × 10^5^ cells from each sample were analysed with three to five mice per group. Purified cells were not used to quantify cell numbers, as significant numbers of DCs are lost during the process of isolation. For studies to determine activation of DCs by expression of MHC class II, CD80, CD86, or for closed examination of subpopulations, cells were purified as described above and then labelled for flow cytometry [61]. To minimize nonspecific labelling, DCs were always preincubated with purified rat Ig in 5% BSA/PBS for 20 min prior to cell labelling, and monoclonal antibodies used were directly conjugated with fluorescein (FITC), phycoerythrine (PE), or allophycocyanin (APC). DCs were routinely analysed for MHC class II (M5/115.15.2), CD80 (16–10A1), CD86 (GL1), CD11c (HL3), CD4 (GK1.5), and CD8 (H35–17.2) expression using reagents purchased from Pharmingen/Becton Dickinson (http://www.bd.com/) and PDCA-1-APC purchased from Miltenyi Biotec. Samples were analysed on FACS Calibur (BD Biosciences, http://www.bdbiosciences.com/), gating on viable cells, using CELLQuest software (version 3.3, BD Biosciences). Approximately 10^4^−2 × 10^5^ cells from each sample were analysed for accurate measurement.

### DC–allogeneic T cell mixed lymphocyte cultures.

DCs were isolated from spleens of naïve or infected mice as described above, titrated, and cultured in triplicates with 2.5 × 10^5^ naïve T cells, isolated from BALB/c or OTII mice. The OTII cultures were supplemented with 100 μg/ml ovalbumin (Sigma-Aldrich, http://www.sigmaaldrich.com/). The T cells were isolated using either MACS anti-Thy 1 beads (Miltenyi Biotec) or R&D Systems (http://www.rndsystems.com/) mouse T cell enrichment kit. Both methods gave >98% purity. After 3 d the culture wells were pulsed with ^3^H-thymidine for 18 h, and radioactive uptake measured.

### Serum cytokines.

The levels of cytokines in the serum were measured using the Becton Dickinson inflammation bead array according to manufacturer's instructions.

### IL-12 secretion.

DCs were isolated as described above and cultured on MultiScreen-HA sterile plates previously coated with 10 μg/ml anti-mouse IL-12 (p70) antibody (Pharmingen) overnight, washed, and unbound sites blocked with 5% FCS. We added 200,000 cells to each well in FCS-supplemented medium alone or with 1.0 μM phosphorothioate-modified CpG oligonucleotide, 1,668 (5′-TCC ATG ACG TTC CTG ATG CT-3′) [63] alone or with 10 μg LPS (*Escherichia coli,* Sigma) [63]. After overnight culture at 37 °C, plates were washed with 0.05% Tween-PBS and incubated with 0.5 μg/ml biotin-anti-IL-12 (Pharmingen) followed by AKP-labelled streptavidin (Pharmingen). NBT/BCIP tablets (Sigma) were used to visualize spots.

### Interferon-α secretion.

DCs were isolated as described above, cultured with CpG DNA and poly-I:C as described previously [49,56], and the secretion of α-(type1)-interferon was assessed by ELISA (PBL Biomedical Laboratories, http://www.interferonsource.com/) according to the manufacturer's instructions.

### Protection studies.

CD11c^+^ DCs were obtained from spleens of naïve mice or mice infected with either 10^4^ *P. yoelii YM* (lethal) or 10^5^ *P. yoelii 17XNL* (nonlethal) pRBCs. Mice were treated with 250 μg pyrimethamine (i.p.) daily for 4 d to clear the infection 4 d after a lethal infection or 7 d after a nonlethal infection. The *P. yoelii YM* infections were started 3 d after the *P. yoelii 17XNL* infections so that both groups of mice could be cured and tested at the same time. At day 10, the spleens were digested and DCs isolated using anti-CD11c MACS beads and passed through three to five MACS columns to obtain a very pure preparation of DCs. Approximately 1.5 × 10^7^ DCs were then transfused intravenously to naïve mice as stated in the text. After resting the mice for >15 h, they were infected with a lethal dose of *P. yoelii YM* (10^4^ pRBCs). Mice were followed for 48 d when monitoring was stopped. To determine whether protection was mediated by IL-12, 0.5 mg of rat anti-mouse IL-12p40 (clone C17.8.20) was administered to mice intravenously the morning after the transfer of DCs, approximately 6–8 h before infection. Additional doses of the anti-IL-12 immunoglobulin were given on the morning of the third and sixth day of infection. To control for the effects of immunoglobulin, equivalent amounts of rat IgG were given to control mice at the same time. To confirm that protection was mediated by IL-12 secreted from donor DCs, CD11c^+^ DCs were obtained from spleens of C57BL/6 or IL-12KO mice (on a C57BL/6 background, >10 backcrosses to C57BL/6) infected with 10^5^ *P. yoelii 17XNL* (nonlethal) pRBCs and then drug cured. Approximately 1.5 × 10^7^ DCs were then transfused intravenously to naïve mice. After resting the mice for >15 h, they were infected with a lethal dose of *P. yoelii YM* (10^4^ pRBCs). Mice were followed for 48 d when monitoring was stopped.

### Statistics.

Error bars shown are means + standard error of means. *p*-Values were calculated using the Mann–Whitney nonparametric *t*-test with a two-sided tail on the basis of pooled data from two to four replicate experiments. The survival curves of mice were statistically analysed using a logrank test based on the Mantel–Haenszel test.

## Abbreviations

DC: dendritic cell
IL-12: interleukin-12
KO: knockout
LPS: lipopolysaccharide
pRBC: parasitized red blood cell
TNF: tumour necrosis factor
